# Three complete mitochondrial genomes of freshwater fishes in the genus *Abbottina* (Cypriniformes: Gobionidae)

**DOI:** 10.1080/23802359.2019.1623728

**Published:** 2019-07-10

**Authors:** Yun Chen, Cuizhang Fu

**Affiliations:** Ministry of Education Key Laboratory for Biodiversity Science and Ecological Engineering, Coastal Ecosystems Research Station of the Yangtze River Estuary, Institute of Biodiversity Science and Institute of Eco-Chongming, School of Life Sciences, Fudan University, Shanghai, China

**Keywords:** Cypriniformes, Gobionidae, Gobioninae, *Abbottina*, China

## Abstract

*Abbottina binhi* has been misidentified as *Abbottina rivularis* in China for a long time. In this study, we determined three mitochondrial genomes of *A. rivularis* and *A. binhi* such that these data should contribute to molecular identifications of the two species. The size of new mitochondrial genomes was 16,609 or 16,599 bp with A + T bias of 55.7–56.5% in the base compositions. Our reconstructed phylogeny showed that *A. rivularis* and *A. binhi* formed a sister taxon relationship, and they together were a monophyletic group.

The genus *Abbottina* belongs to Gobionidae, Cypriniformes (Tan and Armbruster 2018), and they are widely distributed in the Vietnam, China, Japan, North Korea, South Korea and Russian Far East, including *Abottina rivularis* and *A. binhi* (He et al. 2017). *Abottina binhi* has been misidentified as *A. rivularis* in China for a long time (Chen 1998). In this study, we determined three mitochondrial genomes of *A. rivularis* and *A. binhi* such that these data should contribute to molecular identifications of the two species.

One specimen of *A. rivularis* (voucher number: FDZM-ARER20170826) was collected from Erguna City, China (50.27°N, 120.18°E), and two specimen of *A. binhi* (FDZM-ABPIG20170926 and FDZM-ABZONGY20170721) from Pingguo County (23.33°N, 107.55°E) and Zongyang County, China (30.69°N, 117.23°E), deposited in the Zoological Museum of Fudan University (FDZM), China. We obtained genomic DNA using muscle tissues by a high-salt method (Miller et al. 1988). Mitochondrial genomes were assembled after the Sanger sequencing.

The size of new mitochondrial genomes (GenBank numbers MK852688-MK852690) was 16,609 or 16,599 bp. The base compositions displayed A + T bias of 55.7–56.5%. The protein-coding genes used ATG and GTG as start codons, and TAG, TAA, TGA, and T–– as stop codons. The length of control regions showed variable size from 928 to 935 bp. The largest gene overlap (7 bp) was observed between ND4L and ND4 genes. The largest gene interval (31 bp) occurred between tRNA^Asn^ and tRNA^Cys^ genes. The patterns of gene arrangements were the same as other published mitochondrial genomes in fishes of the family Gobionidae (Chen et al. 2015; Li et al. 2018).

The Bayesian analyses were used to infer phylogenetic relationships of *Abbottina* fishes and their close relatives (Tang et al. 2011; He et al. 2017) under the software MrBayes (Ronquist et al. 2012). The reconstructed phylogeny showed that *A. rivularis* and *A. binhi* formed a sister taxon relationship, and they together were a monophyletic group (Figure 1).

**Figure 1.**
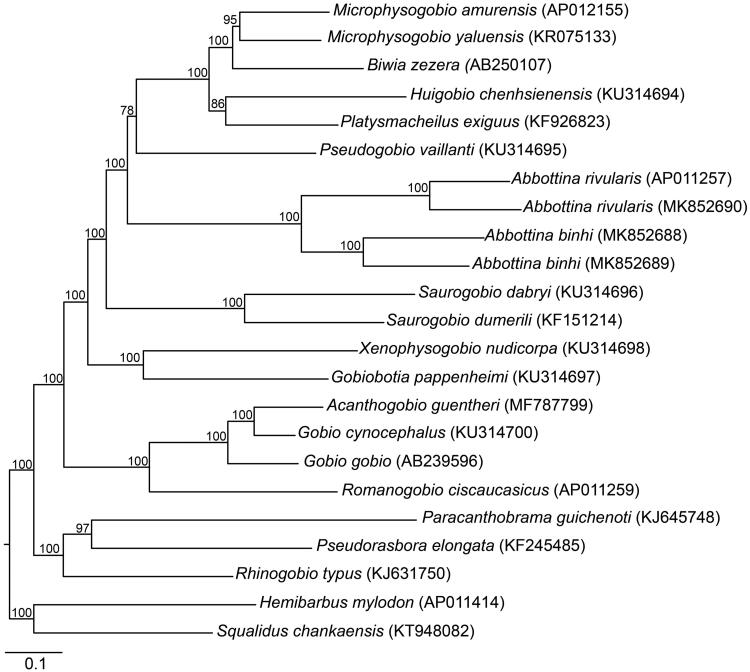
A Bayesian tree of phylogenetic relationships among *Abbottina* fishes and their close relatives based on mitochondrial genomes. The numbers above branches in the phylogeny are Bayesian posterior probabilities. GenBank accessions are listed in the parentheses.
